# *Bacillus amyloliquefaciens* ART9 modulates the growth of *Radix serratulae Chinensis* through rhizosphere microbiome reprogramming and root transcriptomic regulation

**DOI:** 10.3389/fpls.2026.1891133

**Published:** 2026-07-10

**Authors:** Jiaxin Wang, Jinjie Xu, Pengzhong Fu, Jiaxing Li, Bin Tang, Yahan Zhang, Zhixiong Zeng, Yafen Fu

**Affiliations:** 1 Hunan Provincial Key Laboratory of the Traditional Chinese Medicine Agricultural Biogenomics, Changsha Medical University, Changsha, Hunan, China; 2 Hunan Provincial University Key Laboratory of the Regional Characteristic Traditional Chinese Medicine Resources and Ecological Agriculture, Changsha Medical University, Changsha, Hunan, China; 3 Yuelushan Laboratory Aquatic Variety Breeding Center, Hunan Normal University, Changsha, Hunan, China

**Keywords:** *Bacillus amyloliquefaciens* ART9, plant hormone signal transduction, *Radix Serratulae Chinensis*, rhizosphere microbiome, transcriptomics

## Abstract

**Objective:**

Continuous cropping obstacles and heavy chemical fertilizer use constrain *Radix serratulae chinensis* cultivation. This study evaluates the optimal concentration of plant growth-promoting rhizobacterium *Bacillus amyloliquefaciens* ART9 and investigates its underlying mechanisms via rhizosphere microbiome and host transcriptome analyses.

**Methods:**

Seedlings were treated with ART9 suspensions at OD_600_ of 0.4, 0.6, and 1.0. Growth parameters, soil nutrient contents, rhizosphere bacterial communities, and root transcriptomic profiles were assessed to compare treatment effects.

**Results:**

The OD_600_ 0.6 treatment yielded the best performance, increasing plant height, stem diameter, and SPAD value by 48.56%, 14.09%, and 12.42%, respectively, over the control, and raising soil available nitrogen, phosphorus, and potassium by 177%, 179%, and 168%. Rhizosphere microbiota were significantly enriched with beneficial genera, notably *Bacillus, Pseudomonas, and Sphingomonas*. Transcriptomics revealed prominent enrichment in phenylpropanoid biosynthesis (map00940), plant hormone signal transduction (map04075), and circadian rhythm–plant (map04712) pathways. Upregulated genes included *JAZ*, *ABF, PYL, PP2C, SPA1_2*, and *HY5*, indicating coordinated regulation of hormone and light signaling.

**Conclusion:**

ART9 systematically promotes growth of *Radix serratulae chinensis* by reshaping the rhizosphere microbiome and modulating hormone/light signaling pathways, supporting its development as a sustainable biofertilizer for medicinal plant production.

## 1 Introduction

*Radix serratulae chinensis*(syn. *Cimicifuga foetida*) is a medicinal herb whose dried rhizome possesses exceptionally high medicinal value. In traditional Chinese medicine (TCM) clinical practice, *Radix serratulae Chinensis* is commonly used to treat wind-heat headache, toothache, oral ulcers, sore throat, rectal prolapse, and uterine prolapse (Zhang et al., 2024b). Modern pharmacological studies have further shown that *Radix serratulae Chinensis* is rich in two major classes of bioactive compounds—9, 19-cycloartane-type triterpenoid saponins and cinnamic acid derivatives—which exhibit anti-osteoporotic, antiviral, antitumor, antiallergic, and estrogen-like activities (Sun et al., 2017). Despite its medicinal value, traditional cultivation is often hindered by continuous cropping obstacles and soilborne diseases, which compromise yield and quality. Microbial fertilizers, which provide both biocontrol and growth promotion, offer a green and sustainable solution to these challenges, including excessive dependence on chemical pesticides and fertilizers, and inconsistent quality of medicinal materials.

Among microbial agents, plant growth-promoting rhizobacteria (PGPR), as a class of naturally occurring, safe, and highly effective beneficial microorganisms, have demonstrated tremendous potential for advancing the green and ecological cultivation of medicinal plants. PGPR can significantly promote the biosynthesis and accumulation of pharmaceutically active compounds through multiple mechanisms (Santoyo et al., 2016). For instance, via direct actions such as nitrogen fixation, phosphate solubilization, siderophore secretion, and phytohormone synthesis (e.g., indole-3-acetic acid), PGPR optimize root architecture and enhance water and nutrient uptake capacity, thereby providing sustained nutritional support to medicinal plants (Gupta et al., 2024; Abubakar et al., 2023). Previous studies have shown that PGPR inoculation can effectively increase biomass, root length, and dry matter accumulation in a wide range of medicinal plant species, laying the foundation for high-yield cultivation (He et al., 2024).

Importantly, PGPR can markedly enhance the adaptability of medicinal plants to abiotic stresses. Under adverse conditions such as salinity, drought, or heavy metal contamination, PGPR can activate the plant antioxidant enzyme system (e.g., superoxide dismutase, catalase, and ascorbate peroxidase), promote the accumulation of osmotic regulators (e.g., proline and soluble sugars), and modulate ion homeostasis. These actions effectively maintain cell membrane stability and physiological function, ensuring normal growth and secondary metabolism in medicinal plants even under unfavorable environmental conditions (Patani et al., 2023; Dhiman et al., 2026). This property provides a feasible pathway for the ecological cultivation of authentic medicinal herbs on marginal lands. In terms of soil management, PGPR also play an active role: they can promote soil aggregate formation by secreting extracellular polysaccharides, thereby improving water and nutrient retention capacity; certain strains can also reduce the bioavailability of heavy metals through biosorption, metal chelation, and ACC deaminase activity, contributing to the green remediation of contaminated soils (Gupta et al., 2024; Oubohssaine et al., 2025). Furthermore, PGPR can induce systemic resistance in plants, suppress soilborne pathogens, and reduce reliance on chemical pesticides, thus safeguarding the safety and authenticity of Chinese medicinal materials at the source (Jiao et al., 2021; Wang et al., 2024).

Among PGPR, Bacillus amyloliquefaciens is particularly important because of its pronounced growth-promoting and stress-tolerance-enhancing effects in multiple crops. This bacterium secretes active substances such as indole-3-acetic acid (IAA) and spermidine, and induces upregulation of IPT gene expression in plant roots via secreted signaling molecules, thereby increasing endogenous cytokinin (CTK) biosynthesis (Upadhyay et al., 2022). These actions promote seed germination, root development, and chlorophyll synthesis, significantly enhancing biomass and physiological activity in crops such as tomato, rice, and cucumber (Shao et al., 2015). Recent research has progressed from phenotypic observations to molecular investigations. Transcriptomic analyses have revealed that *B. amyloliquefaciens* GKT04, isolated from banana roots, exhibits high inhibitory activity against *Fusarium oxysporum* f. sp. *cubense* race 4 (FOC4), demonstrating its considerable potential for the biocontrol of Fusarium wilt (Tian et al., 2021). *B. amyloliquefaciens* SN16–1 activates defense responses in tomatoes via systemic acquired resistance, which is dependent on the salicylic acid signaling pathway, thereby helping the host resist pathogenic fungi (Zhao et al., 2021). Similarly, in maize and cucumber systems, integrated multi-omics analyses have successfully linked differentially expressed genes with the synthesis of secondary metabolites, elucidating the molecular networks underlying environmental stress responses, environmental sensing, metabolic regulation and host–microbe interactions (Tian et al., 2021; Xu et al., 2020; Oleńska et al., 2020). Nevertheless, single-omics approaches are insufficient to comprehensively resolve the complex regulatory networks that govern PGPR–plant interactions. Given that the growth-promoting effects of *B. amyloliquefaciens* involve multidimensional responses encompassing hormonal regulation, metabolic reprogramming, and immune activation, there is an urgent need to integrate transcriptomic and microbiome datasets at multiple levels to systematically elucidate the underlying growth-promoting mechanisms of this bacterium. Multi-omics integrative analyses not only enable the precise identification of key functional genes and metabolic pathways but also clarify the intrinsic mechanisms governing the synthesis of plant secondary metabolites, stress resistance, and yield formation (Singh et al., 2022; Yang et al., 2022).

Unlike previous studies that merely reported growth promotion by *Bacillus amyloliquefaciens*, the present study offers three distinct novelties: (1) first determination of the optimal application concentration (OD_600_=0.6) of *Bacillus amyloliquefaciens* ART9 for promoting *Radix serratulae chinensis* growth, providing a key parameter for standardized field application; (2) first integration of rhizosphere microbiome and root transcriptome to systematically reveal the cascade regulatory network of ‘strain concentration–rhizosphere microecology–growth response’; (3) first discovery that ART9 promotes medicinal plant growth through coordinated regulation of phytohormone signaling and circadian rhythm pathways, a mechanism not yet reported in related species. This study provides a theoretical foundation and technical support for the development of green cultivation technologies for *Radix serratulae chinensis*, thereby promoting its standardized and industrialized development.

## 2 Materials and methods

### 2.1 Materials and experimental design

The plant growth-promoting strain *Bacillus amyloliquefaciens* ART9 used in this experiment was preserved and supplied by the Key Laboratory of Traditional Chinese Medicine Agriculture, Changsha Medical University. Strain ART9 was molecularly identified by 16S rRNA gene sequencing (GenBank accession: PV174444) and whole-genome draft analysis (BioProject: PRJNA1475832). Phylogenetic analysis showed 99.98% 16S rRNA sequence similarity to Bacillus velezensis reference strain (GCF_001461825.1), with an average nucleotide identity (ANI) of 98.5% to *Bacillus amyloliquefaciens* DSM7T. The strain was cultured to logarithmic phase and adjusted to OD_600_ of 0.4, 0.6, and 1.0, corresponding to CFU of (1.2 ± 0.3)×10^6^, (2.5 ± 0.4)×10^6^, and (4.8 ± 0.6)×10^6^ CFU mL^-1^, respectively. The selected OD_600_ values were based on preliminary experiments: OD_600_<0.4 showed no significant growth-promoting effect, while OD_600_ >1.0 caused excessive cell density potentially leading to competitive inhibition. The strain was aerobically cultured in LB liquid medium at 28 °C with shaking at 180–220 rpm until reaching the logarithmic growth phase (OD_600_ = 0.4–1.0) for subsequent use. Uniform 30-day-old *Radix serratulae chinensis* seedlings were collected from the planting base in Guidong County, Chenzhou City, Hunan Province. After conventional field soil preparation, robust seedlings with consistent growth were transplanted. Root irrigation treatments with bacterial suspensions at OD_600_ values of 0.4, 0.6, and 1.0 were established, with 20 mL applied per seedling in three separate applications; sterile water was used as the control. Each treatment included three independent biological replicates, with 7 uniform seedlings planted within each replicate (21 seedlings in total per treatment). All plants were managed under conventional field conditions without the application of chemical fertilizers or pesticides. After treatment, agronomic traits such as plant height, stem diameter, and root morphology were determined according to previously reported methods to evaluate the growth-promoting effect of the strain.

### 2.2 Determination of agronomic traits and soil physicochemical properties

Plant height and stem diameter were directly measured using a digital Vernier caliper with a precision of 0.01 mm. Leaf SPAD values were measured at the same leaf position using a TYS-4N chlorophyll meter. Soil physicochemical properties were determined using standard laboratory protocols: pH (soil:water ratio 2.5:1, pH meter method); organic matter (potassium dichromate oxidation-external heating method); alkali-hydrolyzable nitrogen (alkaline hydrolysis diffusion method); available phosphorus (sodium bicarbonate extraction-molybdenum antimony anti-colorimetric method); available potassium (ammonium acetate extraction-flame photometry). Each sample was measured in triplicate, and results are presented as mean ± SD.

### 2.3 Rhizosphere soil microbiome sequencing and analysis

Rhizosphere soil samples were collected using the five-point sampling method, with three to six biological replicates per group. Total microbial DNA was extracted using a soil microbial total DNA extraction kit, and DNA integrity and purity were verified by 1% agarose gel electrophoresis and a NanoDrop spectrophotometer, respectively (Versmessen et al., 2024). The V3–V4 hypervariable region of the 16S rRNA gene was PCR-amplified using barcode-labeled specific primers (Kim et al., 2023). Qualified PCR products were used for library construction and subsequently subjected to paired-end sequencing on the Illumina NovaSeq 6000 platform. Raw sequencing reads were quality-controlled and merged using fastp (v0.23.4) and FLASH (v1.2.11), with low-quality sequences filtered out and chimeras removed. Operational taxonomic units (OTUs) were clustered at 97% sequence identity using USEARCH (v11). This 97% OTU threshold was adopted to align with the SILVA 138.2 database annotation standard and facilitate comparison with published PGPR rhizosphere studies. Taxonomic annotation was performed using the RDP Classifier (v2.11) against the SILVA 138.2/16S_bacteria database with a confidence threshold of 70%. After removing chloroplast and mitochondrial sequences, OTU abundance data were rarefied to 49, 248 sequences per sample. Alpha- and beta-diversity analyses were performed using Mothur (v1.30.2), differential species analysis was conducted in R (v3.3.1), and functional prediction was performed using PICRUSt2 (v2.2.0) with default parameters.

### 2.4 Root transcriptome sequencing and qRT-PCR validation of *Radix serratulae Chinensis*

On day 28 after bacterial suspension treatment, three uniformly growing *Radix serratulae chinensis* plants were sampled from the treatment (OD_600_ = 0.6) and control groups. Three biological replicates were set per treatment (each replicate consisted of roots pooled from 7 seedlings). Although the sample size is modest, based on previous transcriptomic studies in medicinal plants (Li et al., 2020), three biological replicates combined with stringent DEG thresholds (|log_2_FC|≥1, FDR<0.05) are sufficient to identify major regulatory pathways. Future studies should increase sample size to validate these findings. Root samples were immediately frozen in liquid nitrogen and stored at −80 °C for total RNA extraction, quality assessment, and Illumina NovaSeq 6000 PE150 sequencing, with ≥6 Gb clean data generated per sample (Modi et al., 2021). Raw reads were aligned to the *Radix serratulae chinensis* reference genome(https://ngdc.cncb.ac.cn/gwh/Assembly/486/show), but the alignment rate was only 0.31%–0.78%; we therefore used Trinity for *de novo* assembly. After quality control of raw sequencing data, Q20≥96.5%, Q30≥91.2%. A total of 28, 490 unigenes were assembled with N50 = 1, 256 bp. The average mapping rate of unigenes back to the transcriptome was 82.3%. BUSCO assessment showed 78.3% completeness. Annotation efficiency: NR 68.7%, KEGG 51.2%, GO 58.3%. Unigenes were annotated against the NR, SwissProt, KEGG, and GO databases. Differentially expressed genes (DEGs) were screened using DESeq2 with thresholds of |log_2_FC| ≥ 1 and FDR < 0.05 (Surachat et al., 2022). Venn diagram and principal component analysis (PCA) were subsequently performed, followed by KEGG enrichment analysis of DEGs related to growth, nutrient absorption, hormone signaling, secondary metabolism, and stress-response pathways, revealing that ART9 activated multiple growth-related pathways and providing candidate genes for further mechanistic investigation (Li et al., 2020). To verify the reliability of the transcriptome sequencing results, key DEGs associated with growth, hormone signaling, and metabolic pathways were selected for qRT-PCR validation. All primer pairs exhibited amplification efficiencies between 90% and 110% (**Supplementary Table 1**), with single-peak melting curves confirming amplification specificity and no primer-dimer formation. Reference genes GAPDH and 18S rRNA were evaluated using geNorm (stability M < 0.5) and used as internal controls (**Supplementary Table 1**). Reverse-transcribed cDNA was used as the template for qRT-PCR analysis. Each sample was run in three technical replicates. Relative gene expression levels were calculated using the 2^-^ΔΔCt method (Lv et al., 2021), and the consistency between qRT-PCR results and transcriptome data was analyzed, thereby providing data support and candidate genes for further elucidating the molecular mechanisms by which ART9 regulates the growth of *Radix serratulae chinensis*.

### 2.5 Integration of phenotypic and microbiome data

To systematically elucidate the coordinated changes among *Radix serratulae chinensis* phenotypic traits, soil physicochemical factors, and rhizosphere microbial community structure, a distance-based redundancy analysis (db-RDA) was employed for multidimensional data integration. Traditional redundancy analysis (RDA) imposes stringent requirements regarding data normality and linear relationships, whereas microbiome data are typically characterized by high dimensionality and sparsity. Db-RDA effectively overcomes these limitations by incorporating a distance matrix, thereby enabling a more accurate quantification of the extent to which environmental factors explain community variation.

### 2.6 Statistical analysis

All experiments were conducted using a completely randomized design. For agronomic phenotype measurements, the mean value of 7 seedlings within each biological replicate was taken as a single data point; final data are presented as mean ± standard deviation (SD) of three independent biological replicates (n = 3). All data were first subjected to Shapiro-Wilk normality test and Levene’s test for homogeneity of variances. Data meeting normality and homoscedasticity were analyzed by one-way ANOVA; otherwise, the Kruskal-Wallis non-parametric test was used. For microbiome differential analysis, the Benjamini-Hochberg method was applied to control the false discovery rate (FDR < 0.05). Following analysis of variance (ANOVA), Tukey’s HSD test was used for multiple comparisons, with statistical significance set at p ≤ 0.05. Data organization was performed using Microsoft Excel, and all statistical analyses and graphical processing were conducted using GraphPad Prism 10.1.2 software based on a one-way ANOVA.

## 3 Results

### 3.1 Concentration-dependent growth-promoting effects of ART9 on *Radix serratulae Chinensis* and soil properties

**Figure 1:**
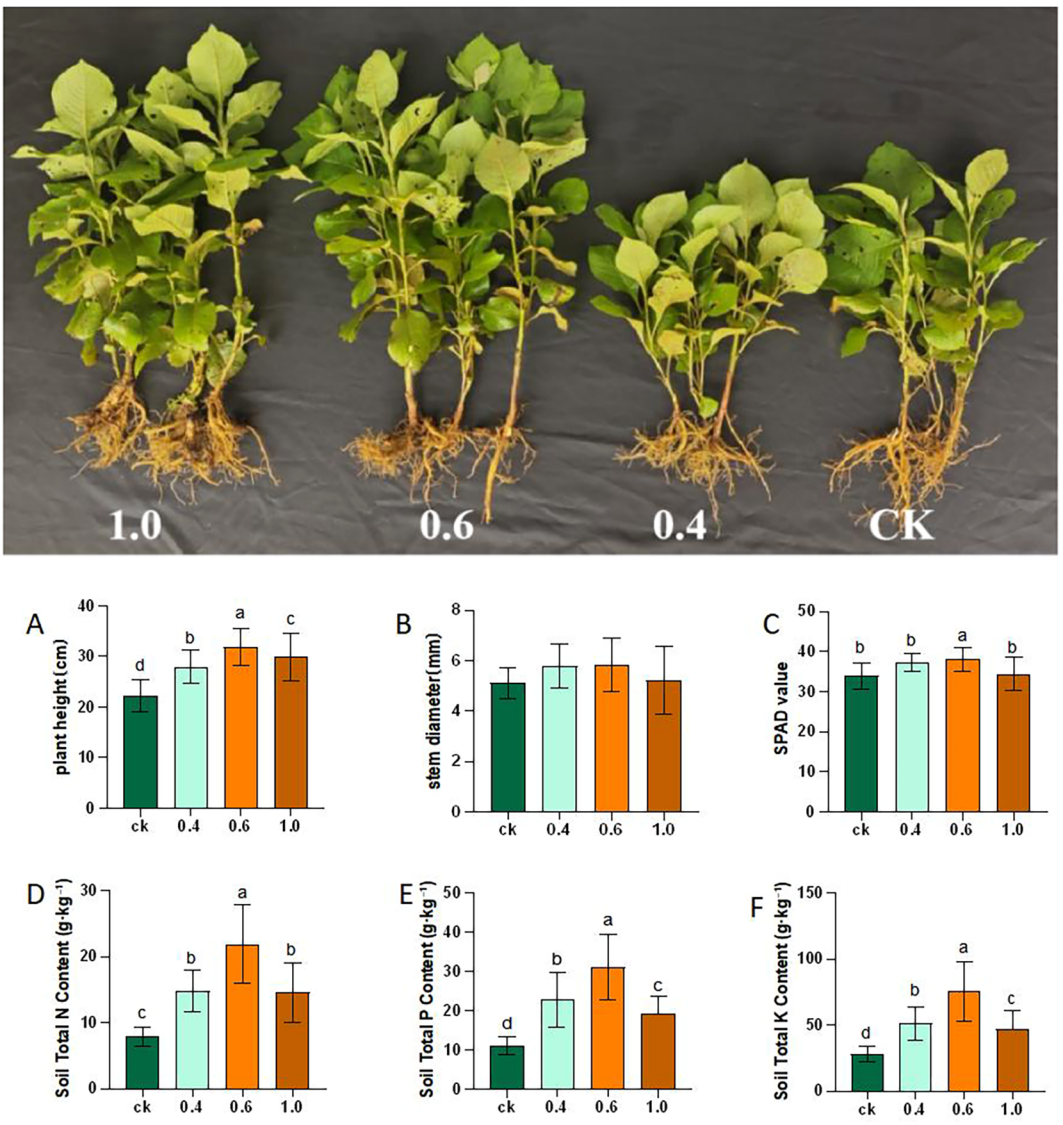
Phenotypic growth of *Radix serratulae chinensis* plants after treatment with *Bacillus amyloliquefaciens* suspensions at different OD_600_ concentrations. Various growth indices of *Radix serratulae chinensis* following treatment with *Bacillus amyloliquefaciens* suspensions at different OD_600_ concentrations: **(A)** plant height; **(B)** stem diameter; **(C)** SPAD value; **(D)** soil nitrogen content; **(E)** soil phosphorus content; and **(F)** soil potassium content. Data are mean ± SD (n = 7 plants per biological replicate, three biological replicates). Different lowercase letters above the bars indicate statistically significant differences among treatments at p ≤ 0.05 (Tukey’s HSD test).

*Bacillus amyloliquefaciens* ART9 was used as the test strain and cultured in LB medium to OD_600_ values of 0.4, 0.6, and 1.0, representing three concentration gradients, to investigate its effects on the growth and development of *Radix serratulae chinensis*. The results showed that all ART9 treatments at different concentrations significantly promoted the growth of *Radix serratulae chinensis*, with the OD_600_ = 0.6 treatment group showing the optimal effect. Growth indices, including plant height, stem diameter, and SPAD values, as well as soil nitrogen (N), phosphorus (P), and potassium (K) contents (**Table 1**), were all significantly higher in the OD_600_ = 0.6 treatment group than in the other treatment groups and the control (CK), demonstrating a clear concentration-dependent peak response (**Figure 1**).

**Table 1:** Effects of *Bacillus amyloliquefaciens* with different OD_600_ values on indexes of *Radix serratulae Chinensis*.

D_600_	Plant height (cm)	Stem diameter (mm)	SPAD	N (mg kg^-1^)	P (mg kg^-1^)	K (mg kg^-1^)
Ck	22.18 ± 3.10	5.11 ± 0.57	33.89 ± 3.18	7.90 ± 1.45	11.10 ± 2.21	28.20 ± 5.38
0.4	28.82 ± 3.34	5.78 ± 0.83	37.18 ± 2.14	14.80 ± 2.96	22.80 ± 6.66	51.20 ± 12.20
0.6	32.95 ± 3.96	5.83 ± 1.00	38.10 ± 2.85	21.90 ± 5.66	31.00 ± 7.95	75.50 ± 21.50
1.0	30.90 ± 4.68	5.22 ± 1.26	34.42 ± 4.00	14.60 ± 4.25	19.20 ± 4.12	46.90 ± 13.52

Data are presented as mean ± SD (n = 3 biological replicates, each with 7 seedlings). Different lowercase letters within the same column indicate statistically significant differences among treatments at *p* ≤ 0.05 (Tukey’s HSD test).

In terms of plant height, the OD_600_ = 0.6 ART9 treatment group attained the maximum value, representing a significant increase of 48.56% compared with that of CK. As the ART9 concentration increased from OD_600_ = 0.4 to 0.6, plant height showed a sustained and significant upward trend; however, when the concentration was further increased to OD_600_ = 1.0, the plant height declined. These results indicate that OD_600_ = 0.6 is the optimal concentration for promoting plant height growth in *Radix serratulae chinensis*. Regarding stem diameter, the OD_600_ = 0.6 treatment group ranked first among all treatment groups, showing a 14.09% increase compared with CK. This indicates that the ART9 bacterial suspension exerts a significant promoting effect on stem diameter in *Radix serratulae chinensis*, with the OD_600_ = 0.6 treatment producing the best results.

The trend in SPAD values, which reflect the relative chlorophyll content of the plant leaves, was consistent with that observed for plant height. The OD_600_ = 0.6 treatment group reached the highest SPAD value, representing an increase of 12.42% compared with that of CK. This result indicates that the OD_600_ = 0.6 ART9 bacterial suspension exerts the strongest promoting effect on chlorophyll synthesis in *Radix serratulae chinensis*, thereby effectively enhancing the photosynthetic potential of the plants.

### 3.2 Response of rhizosphere microbial community structure to ART9 concentration

**Figure 2:**
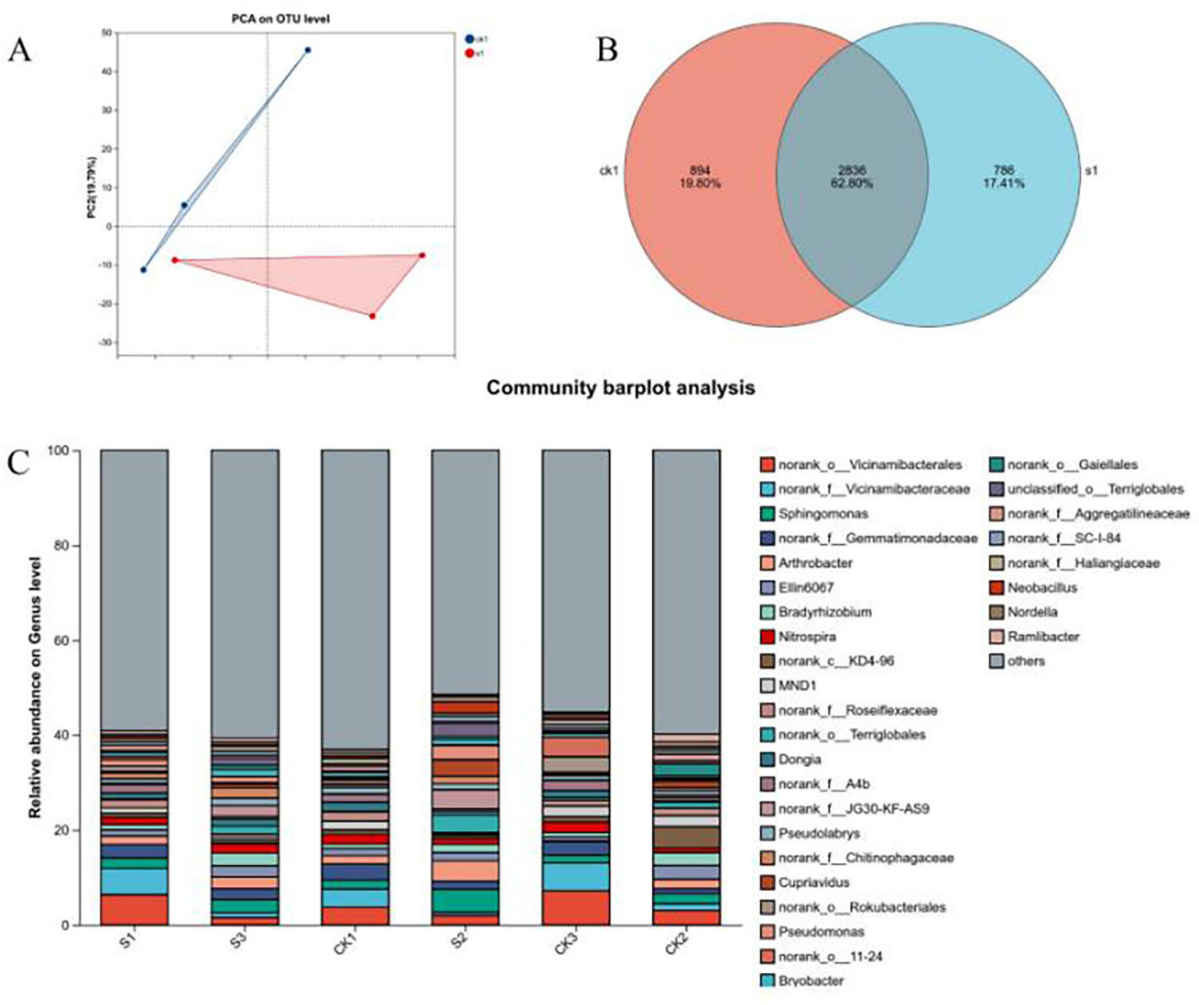
Analysis of rhizosphere soil microbial community characteristics in ART9-treated and untreated *Radix serratulae chinensis*. **(A)** Principal coordinate analysis (PCoA) of rhizosphere soil microbial communities in ART9-treated and untreated groups based on taxonomic classification. **(B)** Venn diagram showing OTU distribution in rhizosphere soil microbial communities between ART9-treated and untreated groups. **(C)** Bar plot analysis of rhizosphere soil microbial community composition in ART9-treated and untreated groups.

Principal coordinate analysis (PCoA) at the OTU level revealed a clear separation trend between the microbial community structures of the control (CK) and treatment (S) groups (**Figure 2**). PC1 and PC2 explained 32.73% and 19.79% of the total variance, respectively, with a cumulative explanatory rate of over 50%. All CK group samples were concentrated in the negative region of the PC1 axis, whereas all S group samples were distributed in the positive region, with no overlap between the two groups. This indicates that ART9 treatment significantly altered the overall microbial community structure. Furthermore, the CK group sample points were tightly clustered, reflecting high within-group stability; although the S group sample points were slightly more dispersed, the between-group differences remained substantially greater than the within-group differences.

Venn diagram analysis further revealed differences in species composition between the two groups (**Figure 2**). A total of 3730 OTUs were detected in the CK group, of which 894 (19.80%) were unique to this group, whereas the S group yielded 3622 OTUs, including 786 (17.41%) group-specific OTUs. The two groups shared 2836 OTUs, accounting for 62.80% of the total OTU count, indicating that most of the core microbial taxa were common to both groups. However, the presence of unique OTUs suggests that ART9 treatment not only resulted in the loss or reduced abundance of certain species present in the CK group but also facilitated the emergence or significant enrichment of new species in the S group, reflecting a treatment-specific response of the microbial community at the species level.

Analysis of the rhizosphere microbial community composition (**Figure 2C**) showed that compared with the CK group, multiple microbial taxa were markedly enriched or stably detected in the ART9 treatment group (**Figure 2**). Specifically, *Sphingomonas*, *Bradyrhizobium*, *Pseudomonas*, *Nitrospira*, Chitinophagaceae, Gemmatimonadaceae, *Cupriavidus*, and the Terrabacteria group were enriched in the S group.

### 3.3 Transcriptional response of *Radix serratulae Chinensis* root gene expression to ART9 treatment

#### 3.3.1 Gene expression profiling of *Radix serratulae Chinensis*

**Figure 3:**
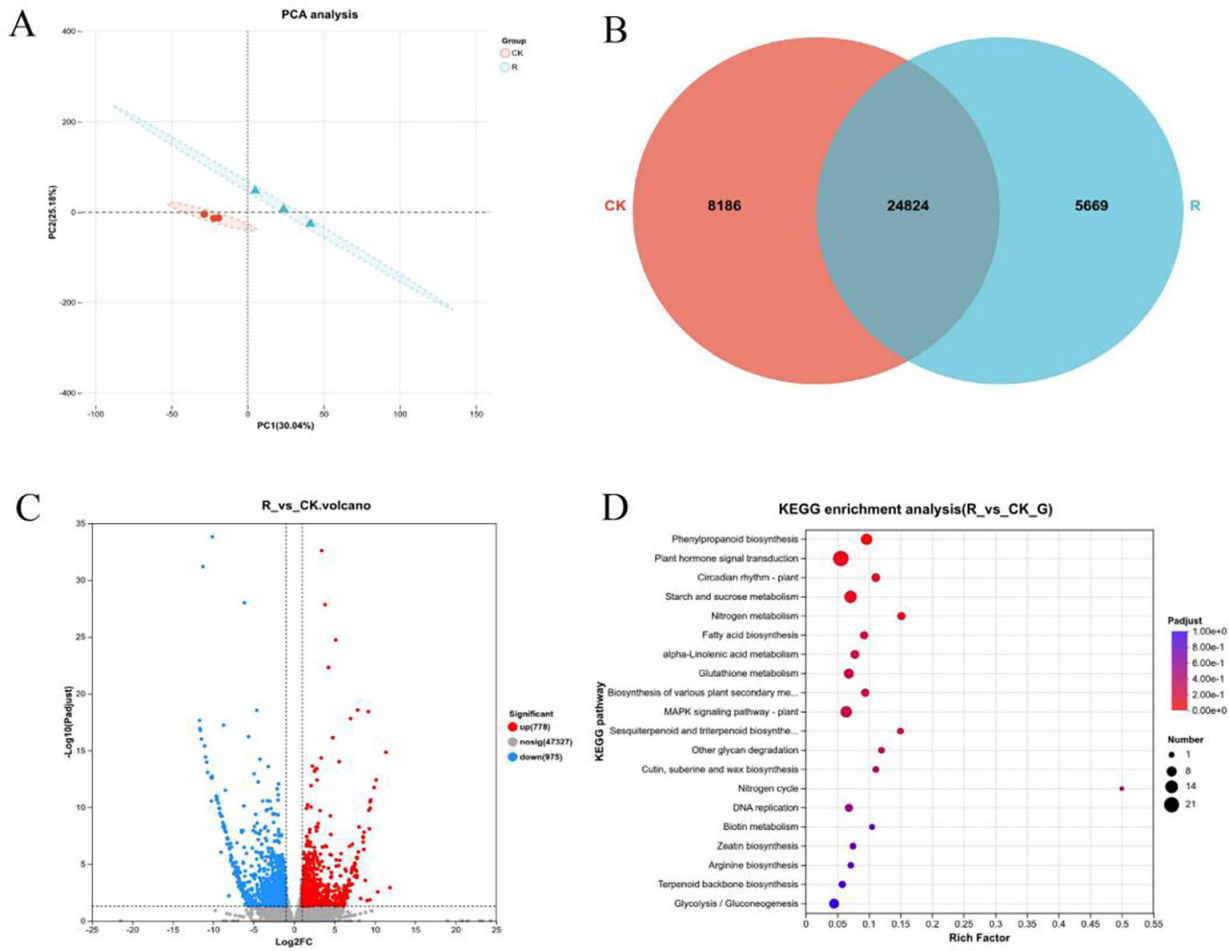
Characterization of root gene expression profiles in ART9-treated and untreated *Radix serratulae chinensis*. **(A)** Venn diagram of root gene expression profiles in ART9-treated and untreated groups. **(B)** Principal component analysis (PCA) of root gene expression profiles in ART9-treated and untreated groups. **(C)** Volcano plot of differentially expressed genes (DEGs) in ART9-treated versus untreated *Radix serratulae chinensis* roots. **(D)** KEGG pathway enrichment scatter plot of DEGs in ART9-treated versus untreated *Radix serratulae chinensis* roots.

To analyze the changes in the gene expression profiles of *Radix serratulae chinensis* following inoculation with *B. amyloliquefaciens* ART9, Venn diagram analysis was first performed to examine the gene distribution between the two groups. The CK group contained considerably more unique genes (8186) than the R group (5669), with a ratio of 1.44:1. The two groups shared 24, 824 genes (**Figure 3A**), indicating that although gene expression exhibited extensive commonality, both groups possessed distinct and group-specific expression profiles. Principal component analysis (PCA) showed that PC1 and PC2 explained 30.04% and 25.18% of the expression variance, respectively. The CK and R group samples were clearly separated in the score plot (**Figure 3B**), confirming that ART9 treatment exerted a significant effect on the gene expression patterns of *Radix serratulae chinensis*, with between-group differences substantially exceeding within-group differences.

Volcano plot analysis was used to identify differentially expressed genes (DEGs). A total of 1753 significant DEGs were identified (|log_2_FC| ≥ 1, p < 0.05), including 778 upregulated genes (44.4%) and 975 downregulated genes (55.6%), with the number of downregulated genes being 1.25-fold higher than that of the upregulated genes (**Figure 3C**). To further characterize the most representative transcriptional changes, key candidate DEGs were summarized in **Table 2**. These results indicate that ART9 inoculation induced widespread transcriptional reprogramming in *Radix serratulae chinensis*, with downregulated genes accounting for a greater proportion than upregulated genes.

**Table 2:** Key candidate genes identified in *Radix serratulae chinensis* roots.

Metabolic pathway	Key upregulated genes	Key downregulated genes
Phenylpropanoid biosynthesis	None	*PAL* (K10775), *CSE* (K12356), *CSE* (K18368), *COMT* (K13066), E2.1.1.104 (K00588)
Plant hormone signal transduction	*PP2C* (K14497), *ARR-B* (K14491), *PYL* (K14496), *ABF* (K14432), *JAZ* (K13464), *YUCCA* (K11816)	*AUX1* (K13946), *ETR* (K14509), *EBF1_2* (K14515), *ERF1* (K14516), *CYCD3* (K14505), *MYC2* (K13422), *TGA* (K14431), *glnA* (K01915), *CALM* (K02183)
Starch and sucrose metabolism	E2.7.1.4 (K00847), *otsA* (K00697), *otsB* (K01087)	*pgm* (K01835), *AMY* (K01176)
Circadian rhythm – plant	*SPA1_2* (K16240), *GI* (K12124), *HY5* (K16241), *FKF1* (K12116)	*CSNK2B* (K03115)

### 3.3.2 Relative gene expression analysis

To further elucidate the growth-promoting mechanisms of *Radix serratulae chinensis* following inoculation with *B. amyloliquefaciens* ART9, KEGG pathway enrichment analysis was performed on the DEGs identified from the transcriptome data. **Figure 3D** presents the top 20 enriched pathways, revealing the metabolic changes induced by ART9 inoculation. Notably, the phenylpropanoid biosynthesis (map00940), plant hormone signal transduction (map04075), and circadian rhythm–plant (map04712) pathways were significantly enriched, with p-values of 0.001, 0.008, and 0.007, respectively. These pathways play critical roles in promoting root cell wall loosening, activating defense responses, and optimizing growth rhythmicity.

In addition, starch and sucrose metabolism (map00500), nitrogen metabolism (map00910), fatty acid biosynthesis (map00061), α-linolenic acid metabolism (map00592), and glutathione metabolism (map00480) pathways were significantly enriched. The coordinated activation of these pathways, all of which exhibited significant upregulation trends, collectively established a comprehensive growth-promoting network encompassing “energy supply–nitrogen utilization–membrane homeostasis–antioxidant defense, ” further highlighting the importance of organic compound synthesis in plant growth. These findings comprehensively elucidate the metabolic reprogramming mechanisms occurring in the roots of *Radix serratulae chinensis* following inoculation with *B. amyloliquefaciens* ART9 and identify the core pathway networks regulating plant growth and development.

### 3.3 qPCR validation

**Figure 4:**
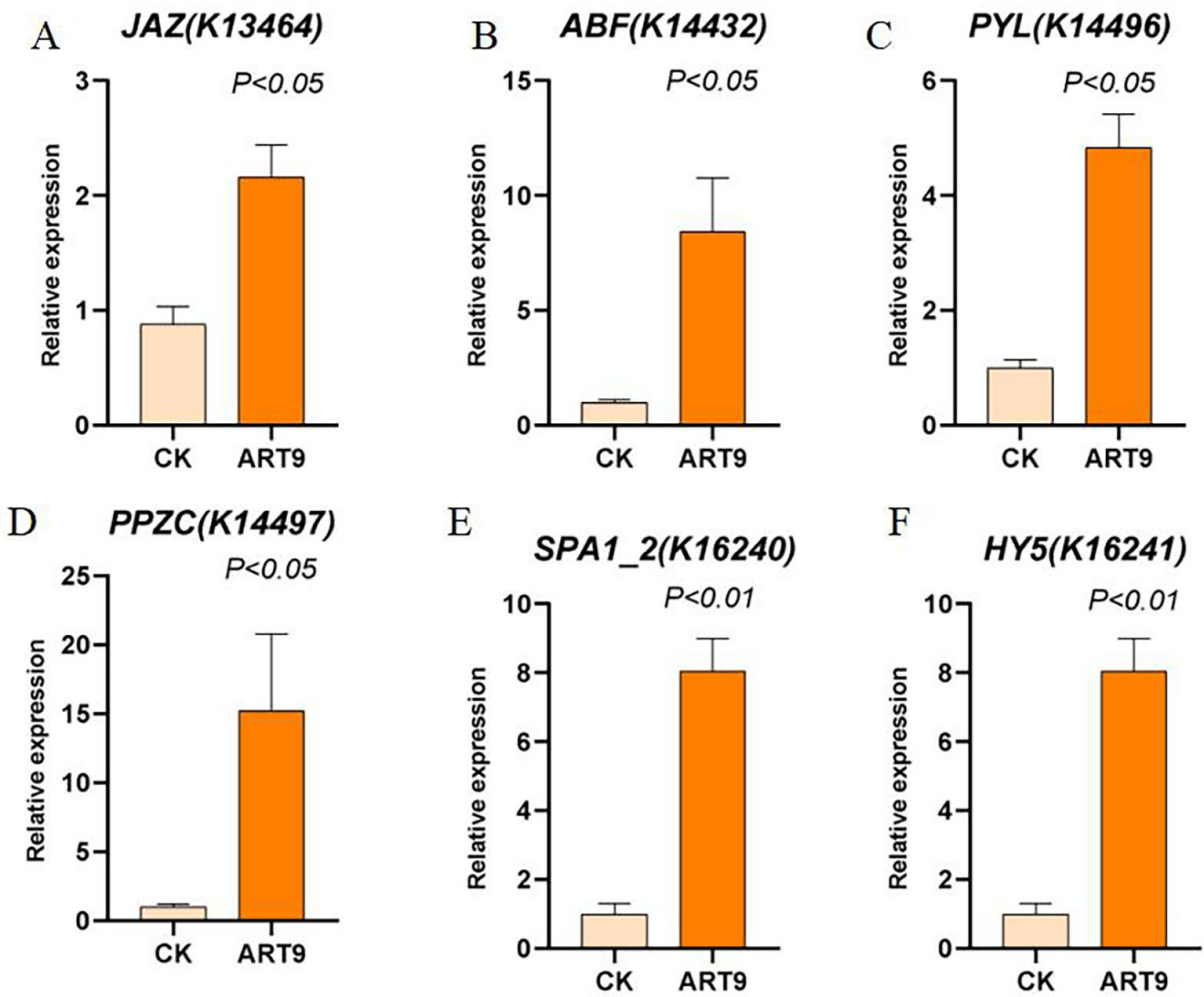
Relative expression analysis of key genes in *Radix serratulae Chinensis* roots following ART9 inoculation. **(A)** Relative expression of JAZ (K13464). **(B)** Relative expression of ABF (K14432). **(C)** Relative expression of PYL (K14496). **(D)** Relative expression of PP2C (K14497). **(E)** Relative expression of SPA1_2 (K16240). **(F)** Relative expression of HY5 (K16241). Data are mean ± SD (n = 3 biological replicates, each with 3 technical replicates).

To further substantiate the growth-promoting mechanisms of *Radix serratulae chinensis* following inoculation with *B. amyloliquefaciens* ART9, qPCR was performed to validate six DEGs identified in the root transcriptome analysis. Compared with the control group, the inoculation treatment group exhibited significantly induced expression of all candidate genes, consistent with the trends observed in transcriptome sequencing data (**Figure 4**).

In the phytohormone signaling pathway, the jasmonate negative regulator *JAZ* (K13464) was upregulated approximately 2.2-fold, whereas the abscisic acid (ABA) signaling components *ABF* (K14432) and *PYL* (K14496) were upregulated approximately 9-fold and 3.5-fold, respectively, indicating the activation of both jasmonate and ABA signaling pathways. Protein phosphatase *PP2C* (K14497) exhibited the most pronounced upregulation, with an approximately 15-fold increase, suggesting its potential involvement in signal transduction through dephosphorylation. In addition, the core light signaling factors *SPA1_2* (K16240) and *HY5* (K16241) were significantly upregulated by approximately 5.3-fold, indicating the involvement of photomorphogenic signaling in the response to inoculation.

The qPCR results validated the reliability of the transcriptome data and confirmed *JAZ*, *ABF*, *PYL*, *PP2C*, *SPA1_2*, and *HY5* as key genes responsive to inoculation. Among these, *PP2C*, *ABF*, *SPA1_2*, and *HY5* were identified as the core regulatory factors. These findings provide an important experimental foundation for elucidating the molecular response mechanisms of *Radix serratulae chinensis* to ART9 inoculation and for the subsequent identification and application of key functional genes in the future.

### 3.4 db-RDA analysis of rhizosphere microbial communities at the OTU level in relation to environmental factor

**Figure 5:**
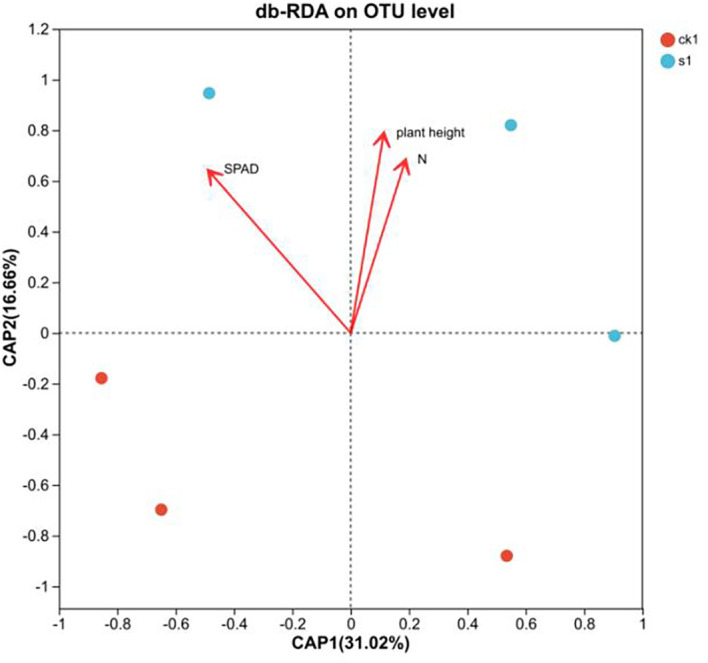
db-RDA analysis of rhizosphere microbial communities and environmental factors in *Radix serratulae Chinensis* at the OTU Level. PERMANOVA analysis showed significant separation between treatment and control groups (*P* < 0.05).

To explore the association between rhizosphere microbial communities and phenotypic traits of *Radix serratulae chinensis*, distance-based redundancy analysis (db-RDA) was performed at the OTU level, and the results are shown in **Figure 5**. The ordination axes CAP1 and CAP2 explained 31.02% and 16.66% of the microbial community variation, respectively, with a cumulative explanatory rate of 47.68%, effectively capturing the major trends in community structure variation.

In terms of sample distribution, rhizosphere soil microbial communities exhibited clear intergroup separation between the PGPR treatment (s1) and control (ck1) groups. Samples from the s1 group were predominantly concentrated in the right region of the ordination space, whereas those from the ck1 group were distributed in the left region, indicating that PGPR treatment significantly altered the bacterial community structure in the rhizosphere soil of *Radix serratulae chinensis*, resulting in marked differences in community composition between the two groups.

Further analysis of the relationships between environmental factors and community structure revealed that the vectors representing plant height and soil nitrogen content (N) were highly consistent with the distribution of s1 group samples, with an extremely small angle between them, indicating a strong and positive correlation. In contrast, the vector representing chlorophyll content (SPAD) was oriented toward the distribution of the ck1 group samples. These results suggest that the divergence in rhizosphere microbial communities induced by PGPR treatment was primarily driven by changes in plant height and soil nitrogen content in *Radix serratulae chinensis*, and that these two factors exerted synergistic effects on community structure. The microbial communities in the control group were more closely associated with environmental factors related to chlorophyll synthesis.

The db-RDA results clearly demonstrated associations between rhizosphere microbial communities and key growth traits of *Radix serratulae chinensis* under PGPR treatment, providing correlative ecological evidence that may help elucidate the mechanisms by which PGPR promotes the growth of *Radix serratulae chinensis* through the regulation of rhizosphere microbial communities. However, these associations do not establish causation.

## 4 Discussion

PGPR are a class of beneficial bacteria that colonize plant roots and promote the growth of medicinal plants, including *Radix serratulae Chinensis*, through diverse direct and indirect mechanisms (Aarti et al., 2024). Screening highly effective PGPR strains and understanding their regulatory mechanisms are essential for advancing green cultivation and ensuring stable, high-quality yields of medicinal materials (Wahab et al., 2024; Zhang et al., 2026; Chen et al., 2023). Although numerous studies have demonstrated that PGPR can enhance crop growth by optimizing soil microbial communities and modulating root gene expression (Miljaković et al., 2020; Li et al., 2021), systematic investigation of the optimal concentration of *Bacillus amyloliquefaciens* ART9 for *Radix serratulae Chinensis* cultivation has been lacking. In the present field experiment, *Radix serratulae chinensis* seedlings treated with ART9 suspensions at OD_600_ = 0.6 exhibited significantly superior vigor compared with the other treatments (**Figure 1**). This study is the first to establish OD_600_ = 0.6 as the optimal application concentration of ART9 for promoting *Radix serratulae Chinensis* growth, thereby filling an important technical gap and providing a reliable basis for standardized inoculant application. These findings may directly guide farmers in applying bacterial irrigation during the seedling or transplanting stages to achieve maximal growth promotion with minimal input, thereby reducing cultivation costs and enhancing economic returns.

In addition to optimizing inoculant concentration, this study explored the molecular basis of growth promotion by analyzing genes involved in plant hormone signal transduction and circadian rhythm regulation. qRT-PCR validated the expression levels of six key genes: *JAZ*, *ABF*, *PYL*, *PP2C*, *SPA1_2*, and *HY5* (**Figure 4**). Among these, *JAZ*, *ABF*, *PYL*, and *PP2C* are upregulated genes involved in the plant hormone signal transduction pathway, whereas *SPA1_2* and *HY5* are critical components of the circadian rhythm–plant pathway. The latter two form a dynamic and phase-complementary signaling module. *HY5* acts as a positive regulator of light signaling and is activated at dawn to promote photosynthetic activity (Wang et al., 2021; Yadav et al., 2020; Jing et al., 2020). Conversely, *SPA1* functions as a negative regulator, accumulating at dusk and suppressing *HY5* activity through protein degradation and phosphorylation, thereby facilitating the transition to nocturnal metabolism. Their synergistic action enables plants to optimally allocate resources across the day–night cycle, balancing growth and stress responses, a mechanism particularly beneficial for *Radix serratulae Chinensis* growth. However, it should be noted that the transcriptomic data in this study reveal correlative changes in gene expression rather than direct causal mechanisms. Future studies should employ genetic manipulation (e.g., CRISPR knockout or overexpression of key genes such as HY5 and PP2C) or chemical intervention (e.g., ABA inhibitors) to validate the functional causality of these genes.

Within the plant hormone signal transduction pathway, the upregulation of *JAZ*, a negative regulator of jasmonic acid (JA) signaling, likely represents a feedback inhibition mechanism that prevents excessive activation of JA-mediated defence responses, thereby optimizing the growth–defence balance, consistent with previous studies (Xu et al., 2024; Li et al., 2025). In the abscisic acid (ABA) pathway, the marked upregulation of the transcription factor *ABF* and the receptor *PYL* indicates that ART9 treatment enhances the capacity of plants for ABA perception and response, thereby contributing to stress tolerance (Fidler et al., 2022). Notably, the protein phosphatase *PP2C* (K14497), a core negative regulator of ABA signaling, was induced approximately 15-fold (Chen et al., 2024). PP2C upregulation (15-fold) appears contradictory to ABA signaling activation. A plausible explanation is that under non-stress conditions, ART9 treatment induces strong negative feedback expression of PP2C, thereby relieving ABA-mediated growth inhibition and promoting plant growth. This represents a typical ‘brake’ mechanism rather than direct activation of ABA signaling. Thus, the observed upregulation of ABF and PYL may reflect enhanced basal activity of ABA signaling, while high PP2C expression represents a balancing negative feedback. This dynamic reprogramming of hormone signaling likely enables plants to adapt to fluctuating environmental conditions (Pan et al., 2024). In the circadian rhythm pathway, the synchronous high expression of *SPA1_2* and *HY5* indicates that ART9 treatment optimizes light adaptability and coordinates growth metabolic rhythms, corroborating previous findings regarding the light signaling regulation of plant development (Mankotia et al., 2024; Chen et al., 2016). Collectively, these results confirm that ART9 specifically activates key genes involved in hormone signaling and circadian rhythm pathways, thereby providing a molecular basis for vigorous *Radix serratulae Chinensis* seedling growth.

Beyond molecular regulation, ART9 application profoundly affected the rhizosphere bacterial community. In the present study, the S group showed marked enrichment of *Sphingomonas*, *Bradyrhizobium*, *Pseudomonas*, *Nitrospira*, Chitinophagaceae, Gemmatimonadaceae, *Cupriavidus*, and the Terrabacteria group, all of which are dominant taxa commonly associated with plant growth-promoting functions (**Figure 2C**) (Floc'h et al., 2021; He et al., 2021; Comeau et al., 2021; Zhang et al., 2024a). At the OTU level, db-RDA revealed a clear separation between the PGPR treatment (s1) and control (ck1) groups, indicating that PGPR inoculation significantly reshaped the rhizosphere bacterial community through rhizosphere colonization, niche competition, and modulation of microbial interaction network (**Figure 5**). Among the environmental factors analyzed, plant height and soil nitrogen content (N) emerged as the key drivers, with their vectors closely aligned with the distribution of s1 samples and a strong correlation between them. These findings suggest that PGPR-enriched bacterial communities directly enhance nitrogen uptake and utilization in *Radix serratulae Chinensis*, thereby promoting plant height growth. Enriched taxa such as *Bradyrhizobium*, *Pseudomonas*, and *Cupriavidus* possess nitrogen-fixing, phosphate-solubilizing, and growth-promoting substance-secreting capabilities, providing a microbiological basis for improved nitrogen transformation and assimilation. However, functional predictions in this study were based solely on PICRUSt2, which is computational inference. To validate the predicted microbial functions, future studies should perform metagenomic sequencing and biochemical assays for key functions (e.g., nitrogen fixation via acetylene reduction assay, phosphate solubilization via molybdenum antimony anti-colorimetric method). In contrast, the control group samples were more closely associated with SPAD values (chlorophyll content), suggesting that in the absence of PGPR treatment, the rhizosphere community was dominated by taxa associated with chlorophyll synthesis and photosynthetic efficiency. Although the ck1 group exhibited relatively high SPAD values, the s1 group achieved superior growth vigor through microbial community restructuring, indicating that PGPR promoted growth not by directly enhancing photosynthesis but by optimizing nutrient uptake and hormone regulation. However, functional predictions in this study were based solely on PICRUSt2, which is computational inference. To validate the predicted microbial functions, future studies should perform metagenomic sequencing and biochemical assays for key functions (e.g., nitrogen fixation via acetylene reduction assay, phosphate solubilization via molybdenum antimony anti-colorimetric method).

In this analysis, db-RDA analysis revealed associations between PGPR treatment promoted the enrichment of specific functional microbial groups, as well as their correlation with key phenotypic traits, such as plant height and nitrogen content. However, these associations do not directly imply causation. This positive community–phenotype association represents an important ecological mechanism underlying PGPR-mediated growth promotion (Cheng et al., 2024). Future studies should isolate the enriched dominant functional strains and conduct single-strain or synthetic community re-inoculation experiments in gnotobiotic systems to verify their individual contributions to plant height and nitrogen uptake, thereby establishing causality and identifying core functional strains and their molecular mechanisms. The cascade regulatory network of strain concentration, rhizosphere microecology, and growth response is proposed to explain the growth-promoting effect.

**Figure 6:**
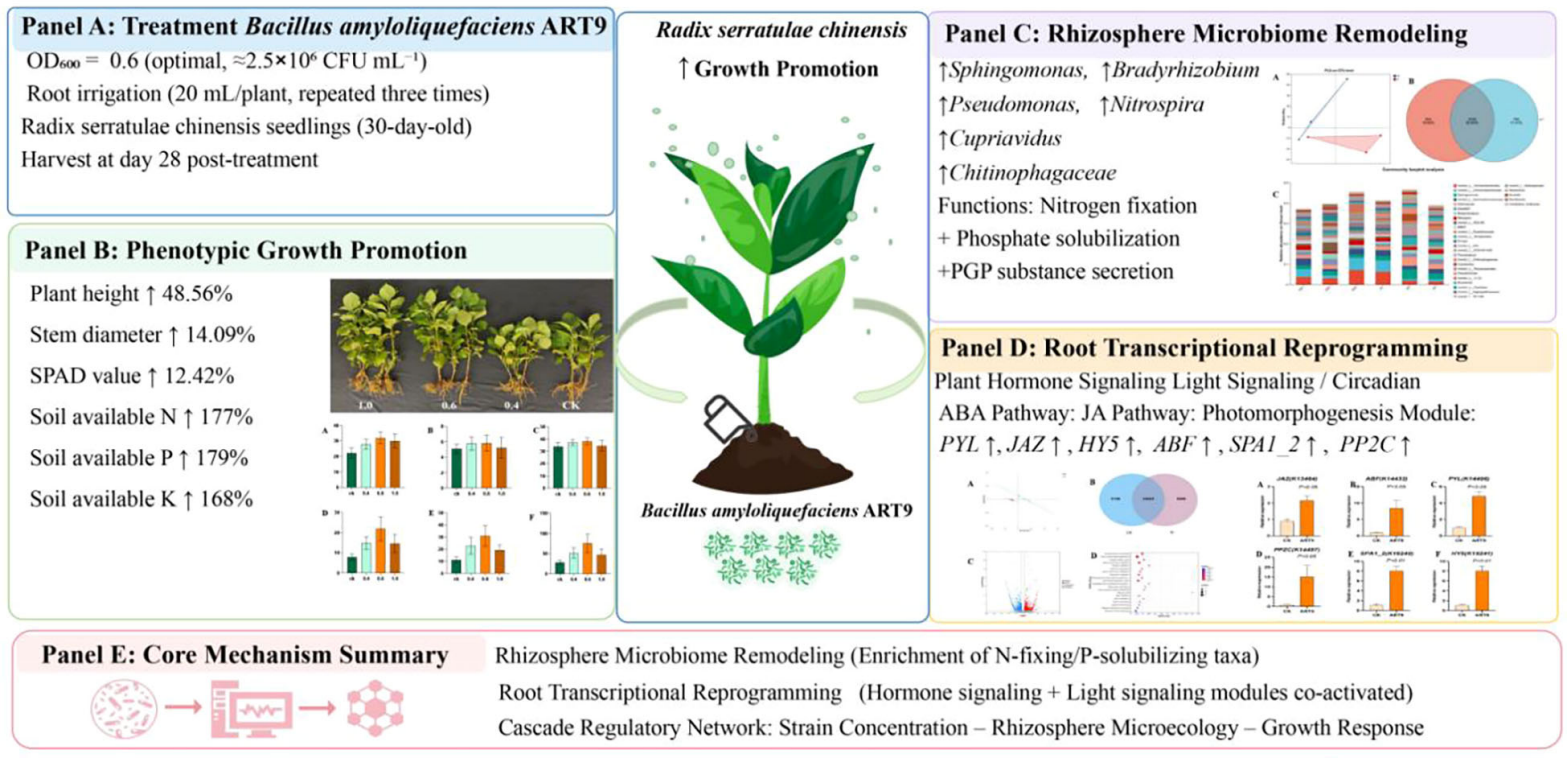
Graphical abstract showing the growth-promoting mechanism of *Bacillus amyloliquefaciens* ART9 on *Radix serratulae chinensis*. **(A)** Thirty-day-old seedlings were root-irrigated with ART9 (OD_600_ = 0.6, 20 mL/plant, three replicates), and samples were collected 28 days after treatment. **(B)** ART9 markedly improved plant growth traits and soil available nitrogen, phosphorus and potassium. **(C)** ART9 reshaped rhizosphere microbiota by enriching multiple beneficial genera responsible for nitrogen fixation, phosphate solubilization and plant growth-promoting metabolite secretion. **(D)** Root transcriptional reprogramming co-activated hormone and light/circadian signaling pathways. **(E)** A cascade regulatory network linking strain dosage, rhizosphere microecology and plant growth response was summarized to explain the growth-promoting effect.

Collectively, this study makes three principal contributions to the literature. First, it identified OD_600_ = 0.6 as a candidate optimal concentration of ART9 for *Radix serratulae Chinensis* growth under the controlled plot conditions tested, providing a theoretical reference for future concentration optimization. Second, it reveals a dual regulatory mechanism at the molecular level, whereby ART9 coordinately modulates plant hormone signaling and circadian rhythm. Third, it integrates rhizosphere microbiome remodeling with phenotypic and transcriptomic data, suggesting that ART9 promotes growth by constructing a nitrogen utilization-centered functional microbial community rather than by directly increasing chlorophyll content (Timofeeva et al., 2022; Kalayu, 2019; Lidbury et al., 2016). **Figure 6** presents a graphical summary of the overall mechanism. As shown in the graphical abstract, ART9 root irrigation at the optimal concentration reshapes the rhizosphere microbiome by enriching N-fixing and P-solubilizing genera (*Sphingomonas, Bradyrhizobium, Pseudomonas, Nitrospira, Cupriavidus, Chitinophagaceae*), which increases soil available nutrients. This microbiome remodeling is coupled with root transcriptional reprogramming that co-activates hormone signaling, including the ABA pathway (*PYL, ABF*, and *PP2C* as a feedback “brake”) and the JA pathway (*JAZ*), as well as light and circadian modules (*HY5* and *SPA1_2*), ultimately promoting plant growth through a cascade regulatory network of “strain concentration- rhizosphere microecology-growth response”. The present study was carried out under controlled field plot experimental conditions to preliminarily explore the growth-promoting mechanism of strain ART9 on *Radix serratulae chinensis*. The current findings provide fundamental theoretical data and technical reference for subsequent large-scale field verification, strain agent development, and field application trials. Further multi-location, multi-year field validation is urgently required before agricultural popularization and commercial utilization.

Nevertheless, several limitations of this study should be acknowledged. The optimal application strategy requires further validation across different soil types, climatic conditions and growth stages. The specific molecular signals through which ART9 regulates host gene expression, either via secreted metabolites or alterations in the rhizosphere microenvironment, remain unclear. In addition, this study did not perform metabolomic analysis; therefore, statements regarding ‘metabolic reprogramming’ are primarily based on transcriptomic inference. In addition, this study focused on non-destructive growth parameters (plant height, stem diameter, and SPAD values) and did not collect destructive samples for biomass measurement (fresh weight and dry weight), which are important indicators for comprehensively evaluating growth-promoting effects. Future studies should include biomass measurements to provide a more complete assessment of ART9-mediated growth promotion. Furthermore, since *Radix serratulae chinensis* is a medicinal plant whose value depends primarily on the content of bioactive constituents (e.g., 9, 19-cycloartane-type triterpenoid saponins and cinnamic acid derivatives), this study focused only on growth promotion and did not quantify these active compounds. Moreover, the individual roles and interaction networks of enriched microbial taxa require experimental verification. To address these gaps, future studies should include multi-site and multi-year field trials, isolation and re-inoculation of dominant strains, identification of signaling molecules in sterile systems, untargeted metabolomics to directly detect changes in metabolites within the phenylpropanoid biosynthesis pathway (e.g., cinnamic acid, coumaric acid, monolignols), HPLC or LC-MS/MS quantification of key pharmacologically active components to assess whether ART9 treatment genuinely improves medicinal material quality and integrated transcriptomic–metabolomic analyses to comprehensively elucidate the global response network of *Radix serratulae Chinensis* roots.

## 5 Conclusions

This study systematically investigated the growth-promoting effects of *Bacillus amyloliquefaciens* ART9 on Radix serratulae chinensis and elucidated the underlying mechanisms through integrated microbiome and transcriptomic analyses. The optimal concentration of ART9 (OD_600_ = 0.6) was identified, which significantly enhanced plant height, stem diameter, chlorophyll content, and soil available N, P, and K. Multi-omics analyses revealed that ART9 reshaped the rhizosphere microbiome by enriching N-fixing and P-solubilizing genera (*Sphingomonas, Bradyrhizobium, Pseudomonas, Nitrospira, Cupriavidus*, and *Chitinophagaceae*), while root transcriptional reprogramming co-activated hormone signaling (ABA and JA pathways) and light/circadian modules (HY5 and SPA1_2). A cascade regulatory network of strain concentration-rhizosphere microecology -growth response is proposed to explain the growth-promoting effect. These findings provide a theoretical foundation and technical reference for the development of green cultivation technologies for *Radix serratulae* chinensis, though further multi-location, multi-year field validation is required before agricultural application.

## Data Availability

The raw sequencing data generated in this study have been deposited in the NCBI database under the following BioProject accession numbers: PRJNA1467762 (16S rRNA gene amplicon sequences), PRJNA1467520 (raw transcriptome data), and PRJNA1475832 (whole-genome draft of strain ART9).

## Supplementary material

Supplementary Table 1 qRT-PCR primers sequences used for validation of differentially expressed genes in *Radix serratulae chinensis* roots.

Supplementary Table 2 KEGG pathway enrichment analysis of differentially expressed genes in Radix serratulae chinensis roots following ART9 treatment.

Supplementary Table 3 Expression profiles and functional annotation of differentially expressed genes in *Radix serratulae chinensis* roots following ART9 treatment.

Supplementary Table 4 OTU abundance table of rhizosphere microbial communities in ART9-treated and control groups.
