# Supplementary material for: Bacillus amyloliquefaciens ART9 modulates the growth of Radix serratulae Chinensis through rhizosphere microbiome reprogramming and root transcriptomic regulation
Source: Front Plant Sci. 2026 Jul 10;17:1891133. doi: 10.3389/fpls.2026.1891133 (PMC13395933; doi:10.3389/fpls.2026.1891133)
Supplement: Supplementary Table 1 — qRT-PCR primers sequences used for validation of differentially expressed genes in Radix serratulae chinensis roots. [file Table1.docx]

### Supplementary Tables S1. qRT-PCR primer sequences used for validation of differentially expressed genes in *Radix serratulae chinensis* roots.

| Gene name | Gene ID (KEGG) | Primer sequence (5′→3′) | Product length (bp) | Annealing temp. (°C) | Amplification efficiency (%) | R² |
| --- | --- | --- | --- | --- | --- | --- |
| JAZ | K13464 | F: TGGGCTACATCAAGCAGATC R: CTTCCATTCGTCACCTTCTG | 120 | 59 | 98.6 | 0.998 |
| ABF | K14432 | F: AACTGCTGGTGGTGATGATG R: GCTTGTTGTTGCTGGTGATG | 115 | 60 | 102.3 | 0.997 |
| PYL | K14496 | F: GGAGCTGCATATCGCTCTTC R: CAACCAACCCGTTACTGTTG | 108 | 59 | 95.7 | 0.996 |
| PP2C | K14497 | F: TGGGAGTTGGTGGTTTCTTC R: GACCTTTGCTTTGCACTTCC | 132 | 60 | 99.2 | 0.999 |
| SPA1_2 | K16240 | F: CGGAGATCGAGAGTGTGAAG R: TGACGACGAAGACTTGATGG | 125 | 58 | 97.8 | 0.997 |
| HY5 | K16241 | F: GCAGTCGTCGTCGTCGTC R: CGGATCCCACTAGTTGCTTC | 95 | 60 | 101.5 | 0.998 |
| β -actin | – | F: CATCCGTAAAGACCTCTATGCCAAC  R:ATGGAGCCACCGATCCACA | 110 | 59 | 99.0 | 0.98 |
